# Melatonin-Induced Transcriptome Variation of Rapeseed Seedlings under Salt Stress

**DOI:** 10.3390/ijms20215355

**Published:** 2019-10-28

**Authors:** Xiaoyu Tan, Weihua Long, Liu Zeng, Xiaoyu Ding, Yong Cheng, Xuekun Zhang, Xiling Zou

**Affiliations:** 1 Key Lab of Biology and Genetic Improvement of Oil Crops of Ministry of Agriculture and Rural Affairs, Oil Crops Research Institute of the Chinese Academy of Agricultural Sciences, Wuhan 430062, China; tanxiaoyu@caas.cn (X.T.); jaaslongweihua@outlook.com (W.L.); zengliu0929@163.com (L.Z.); dingxiaoyu1991@163.com (X.D.); 13808614864@139.com (Y.C.); zhang.xk@139.com (X.Z.); 2 College of Plant Science and Technology of Huazhong Agricultural University, Wuhan 430070, China; 3 Key Lab of Cotton and Rapeseed (Nanjing) of Ministry of Agriculture and Rural Affairs, Institute of the Industrial Crops, Jiangsu Academy of Agriculture Sciences, Nanjing 210014, China

**Keywords:** melatonin, salt stress, *Brassica napus* L., seedling stage, transcriptome variation, hormone

## Abstract

Salt stress inhibits the production of all crop species, including rapeseed (*Brassica napus* L.), the second most widely planted oil crop species. Although melatonin was confirmed to alleviate salt stress in rapeseed seedlings recently, the mechanism governing the expression levels remains unknown. Therefore, the melatonin-induced transcriptome variation of salt-stressed seedlings was explored. In this study, the transcriptomes of leaves and roots under control (CK), salt (125 mM NaCl, ST) and melatonin (125 mM NaCl plus 50 µM melatonin, MS) treatments were evaluated by using next-generation sequencing techniques. After conducting comparisons of gene expression in the roots and leaves between MS and ST, the differentially expressed gene (DEG) pools were screened. Kyoto Encyclopedia of Genes and Genomes (KEGG) enrichment analyses highlighted the significant pathways, which were mainly related to plant hormone synthesis and signal transduction, lignin and fatty acid metabolism. The functional genes in the objective KEGG pathways were identified. Furthermore, members of several transcription factor (TF) families participated in the response process. Combined with the hormone (campesterol (CS), jasmonic acid (JA), and gibberellic acid 3 (GA3)) contents measured in the seedlings, it could be concluded that melatonin induced changes in the intrinsic hormone metabolic network, which promoted seedling growth. Thus, this study identified new candidate genes and pathways active during the interactions between melatonin and salt stress, which provide clues for disclosing melatonin’s function in resistance to salt injury. Our results contribute to developing a practical method for sustainable agriculture on saline lands.

## 1. Introduction

Salt stress is one of the major problems in the soil and leads to a dramatic decrease in global crop production [1]. More than 800 million hectares of land are severely threatened by high salinity. What is more serious is that this tendency seems to be worsening [2]. Much research attention has focused on understanding the mechanisms by which crops counter salt stress and improve their resistance level [3,4,5]. The consistent outcomes of salt tolerance mechanisms are primarily as follows. First, osmolyte accumulation pathways are triggered to balance the high osmotic pressure from outside of the cells. Several classes of compatible osmolytes, including charged metabolites (such as proline and glycine betains), polyols (such as mannitol and glucosyglycerol), soluble sugars (such as sucrose) and complex sugars (such as raffinose), increase under the regulation of their synthesis-direction pathways [5,6]. The synthesis of some osmolytes is regulated by the Mitogen Activated Protein Kinase (MAPK) pathway and ABA signals [7,8,9]. Second, ion homeostasis regulation is activated to cope with the excessive amount of ions inside the cells. High concentrations of ions (Na^+^, Cl^−^, etc.) outside enter cells through ion channels by active and passive absorption, which disrupts the ion balance inside [10,11]. Some important ion transport systems (nonselective cation channels, the salt overly sensitive pathway, etc.) have been found, and key genes have been proven to function in ion efflux [12,13,14,15]. Other ions (K^+^, Mg^2+^, Ca^2+^, etc.) also affect the Na^+^/Cl^-^ influx or efflux [7]. Third, reactive oxygen species (ROS) detrimental to proteins, lipids, DNA and carbohydrates are induced by salt stress and enzyme systems are triggered to eliminate ROS and to repair damaged components. These enzymes include superoxide dismutase (SOD), ascorbate peroxidase (APX), and catalase (CAT) [6,16]. Furthermore, other pathways (such as the ABA-dependent/independent pathway and the calmodulin pathway) linking the metabolism of the above substances also affect the strength of salt stress resistance [17,18]. Genetically, many genetic loci in plant genomes have been identified as having functions in alleviating salt stress [19]. In spite of the significant progress in the understanding of salt tolerance mechanism, crops with the satisfactory salt tolerance are still in need [20,21]. Fortunately, the transcriptomics studies could provide immense data on gene expression at the mRNA level and have achieved a great development recently [22,23]. It will promote the understanding of molecular basis of salt tolerance mechanisms.

Melatonin (*N*-acetyl-5-methoxytryptamine), first discovered in the bovine pineal gland in 1958, acts as a neurohormone contributing to the regulation of many physiological events and as an antioxidant in scavenging free radicals [24]. Although it was found in higher plants in 1995, research has shown that, in plants, melatonin is a molecule with pleiotropic effects on physiology and can be an antistress agent when facing abiotic stresses, including drought, salinity, freezing, toxic chemicals, pathogen attack, and so on [24,25]. Moreover, melatonin exhibits cross-talk with plant hormones during organ growth (such as rhizogenesis) and photosynthesis [24]. These findings suggest it is advantageous for crop improvements. Specifically, several publications have identified a role of melatonin in weakening the damage from salt stress on crops. In soybean, melatonin promotes plant growth, seed production and salt plus drought stress tolerance, and the responsive genes are enriched in the pathways of photosynthesis, carbohydrate metabolism and anti-oxidative actions [26]. In cucumber, exogenous melatonin enhanced seed germination under 150 mM NaCl treatment. The activities of antioxidant enzymes including SOD, CAT, and POD increased several-fold; suggesting melatonin functions in optimizing antioxidant systems and affects the expression of ABA and GA biosynthesis genes [27]. In rice, melatonin acts as a potent agent in delaying leaf senescence and cell death under salt stress. Transcriptome analysis showed that melatonin upregulates genes related to oxidation-reduction, chlorophyll biosynthesis and stress responses [28]. Despite these results, the detailed promotion mechanism of melatonin during the salt stress response process in crop species still needs further investigation.

Rapeseed (*Brassica napus* L., AACC, 2*n* = 38) is a vital oil crop species with the second largest annual planting area and production worldwide [29]. Like other crop species, rapeseed is greatly hindered by salt stress [30]. Generally, the improvement in rapeseed salt tolerance has received limited progress in recent years, despite some publications [31]. Fortunately, exogenous applications of low-concentrate melatonin were proven to restore rapeseed growth strength under salt stress in hydroponic culture [32]. In other words, melatonin could be an enhancer of resistance to high-salt environments, although the mechanism of melatonin action is still unknown. Based on this phenomenon, the objectives of this study were to: (1) explore the gene expression profile of rapeseed seedlings in response to salt and melatonin treatments at the transcriptome level; (2) reveal the regulatory network and confirm the candidate pathways/genes driven by melatonin under salt stress.

## 2. Results

### 2.1. Phenotypes of Rapeseed Seedlings Under Control (CK), Salt (125 mM NaCl, ST) and Melatonin (125 mM NaCl Plus 50 µM Melatonin, MS) Treatments

**Figure 1:**
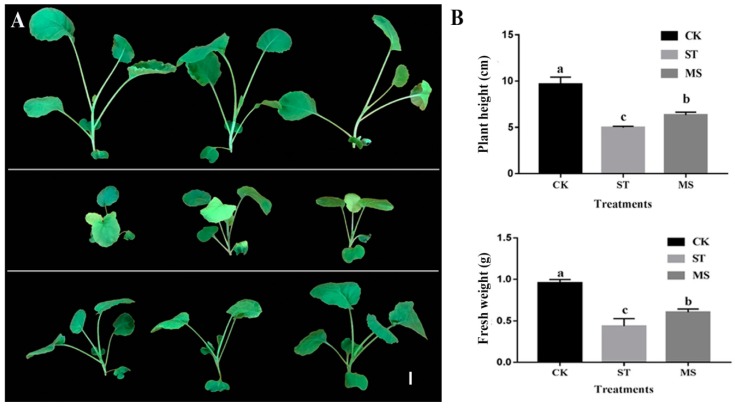
Phenotypes of seedlings under different treatments. (**A**) The upper, middle, and lower rows show seedlings on the 7th day after CK, ST, and MS treatments, respectively. Bar = 1 cm. (**B**) Comparisons of plant height (**upper-right**) and fresh weight (**lower-right**) among the treatments. Experiments were repeated three times and vertical bars indicate standard errors. CK = Control; ST = 125 mM NaCl; MS = 125 mM NaCl plus 50 µM melatonin. The a, b, c on the columns represent significant differences at *p* < 0.05.

Rapeseed seedlings responded differently under the CK, ST, and MS treatments (Figure 1). Compared with the seedlings under the CK treatment, the seedlings under the ST treatment had smaller leaves and significantly decreased fresh weight. However, the seedlings under the MS treatment showed larger leaves and higher fresh/dry weight than did those under ST, which were not the same as those under CK. Thus, melatonin alleviated the symptoms of salt stress in rapeseed seedlings.

### 2.2. Hormone Contents in the Shoots under Different Treatments

**Figure 2:**
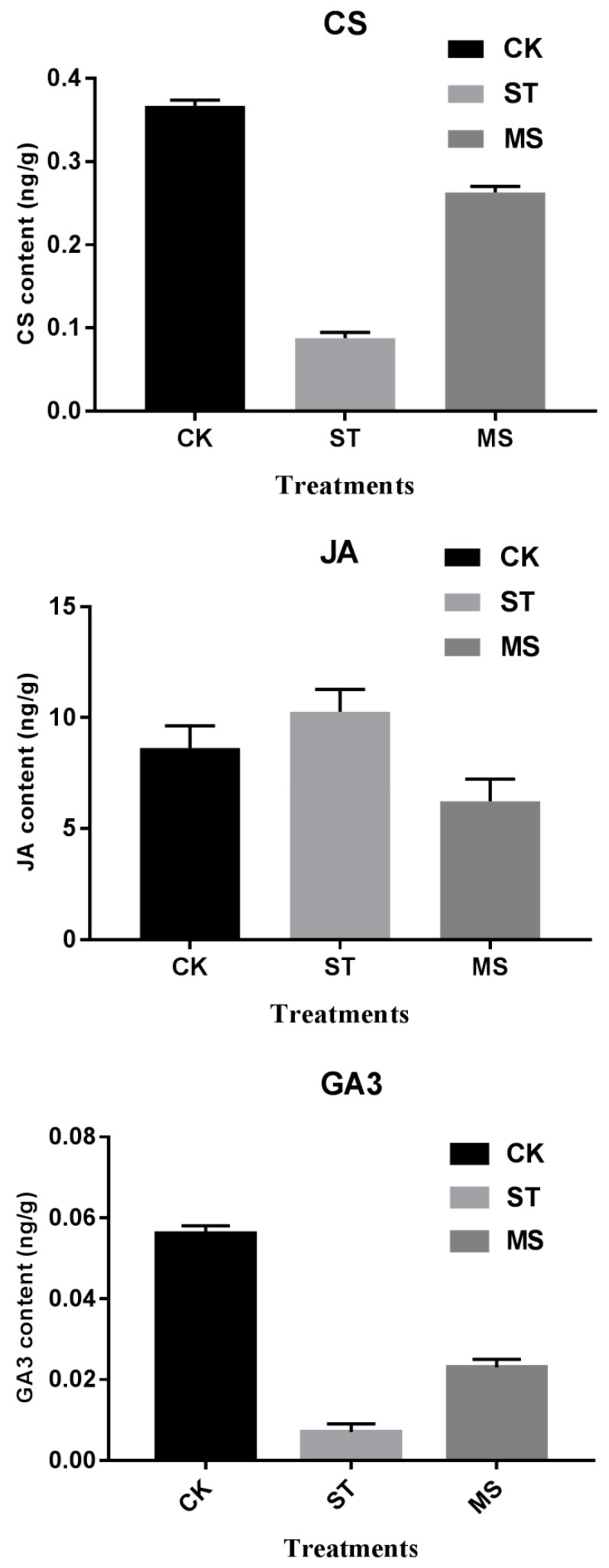
Contents of three hormones in seedling shoots under CK, ST, and MS treatments. CS = campesterol; JA = jasmonic acid; GA3 = gibberellic acid 3. Experiments were repeated three times, and vertical bars indicate standard errors.

Three plant hormones (CS, JA, and GA3) were measured in the shoots under the CK, ST and MS treatments (Figure 2). Compared with that under CK, the JA content under the salt stress treatment increased by 27%; moreover, the CS content decreased fourfold, and the GA3 content decreased sevenfold. Under the MS treatment (with melatonin added to the salt stress treatment), the CS content was restored to 70% of that of the CK, and the GA3 content was restored to 40% of that of the CK, while the JA content decreased to 70% of that of the CK. Therefore, melatonin affected the metabolism of these hormones during salt stress.

### 2.3. RNA Sequencing and Data Analysis

In total, 18 cDNA libraries (three replicates for the leaves and roots under CK, ST and MS) were successfully constructed. The average data for different tissues and treatments are shown in Table 1. There was a total of 6.75 to 9.56 Gb of data, which equaled 44.4 to 62.9 million valid reads, generated for the samples. The reads were mapped to the reference genome, and the proportion (~68%) was good. Each treatment (including the CK) was associated with 39.5 to 47.6 thousand genes (transcripts) via Cufflinks. In total, 99,645 theoretical genes (transcripts) were identified by comparing all the transcripts from all the libraries via the Cuffcomerge module; these genes were set as the basal gene files for subsequent expression analyses.

### 2.4. Gene Expression Descriptions Under the Different Treatments

**Figure 3:**
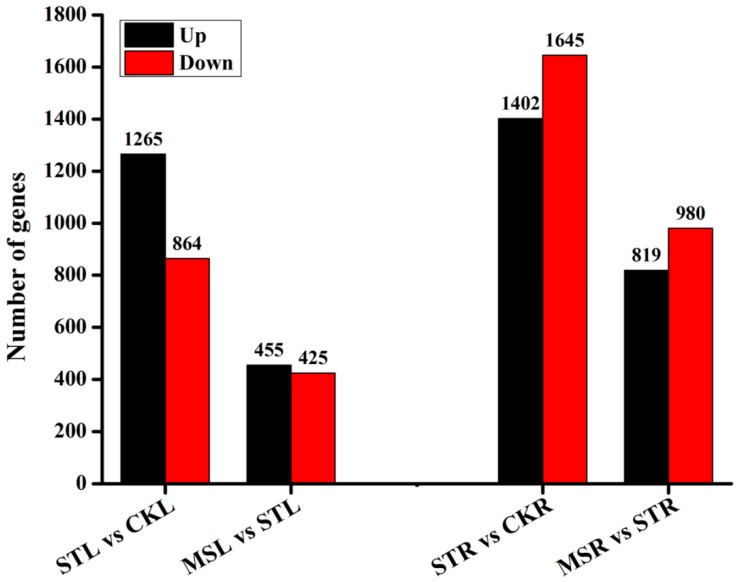
Number of regulated genes in the different comparisons. STL = leaves in ST treatment; CKL = leaves in CK treatment; MSL = leaves in MS treatment; STR = roots in ST treatment; CKR = roots in CK treatment; MSR = roots in MS treatment. The Arabic numbers on the column are the genes numbers of different comparison of leaves/roots between treatments.

Given the thresholds of *q* value ≤ 0.05 and |log_2_ (FPKM (fragments per kilobase of transcript per million mapped reads) ratio)| ≥ 1, the differentially expressed genes (DEGs) were identified by comparing the gene expression strength (in FPKM) between each pair combination of the CK, ST and MS treatments. Four pairwise comparisons were conducted, and the results are shown in Figure 3. For the leaf samples, there were totals of 2149, 2120 and 880 DEGs in the comparisons of STL versus CKL, MSL versus CKL and MSL versus STL, of which 1265, 1185, and 455 DEGs were upregulated and 864, 935, and 425 DEGs were downregulated, respectively. Similarly, for the root samples, there were 3047, 3045, and 1799 total DEGs in the comparison of STR versus CKR, MSR versus CKR and MSR versus STR, of which 1405, 1454, and 819 DEGs were upregulated and 1645, 1591, and 980 DEGs were downregulated, respectively. Two points could be proven from the data: (1) In general, there were more DEGs in the roots were than in the leaves, and (2) there were more upregulated DEGs in the leaves than downregulated DEGs; however, the situation was the opposite in the roots.

Venn diagrams were constructed to show the numbers of DEGs in the treatment comparisons (Figure S1). In the leaves, 1937 DEGs responded specifically to the salt treatment, 688 DEGs responded specifically to the melatonin treatment, and only 192 DEGs responded to the two treatments at the same time. Similarly, in the roots, there were 2667 DEGs that specifically responded to the salt treatment, 1419 DEGs that specifically responded to the melatonin treatment, and 380 DEGs that responded to the two treatments at the same time. Generally, 631 DEGs were specifically stimulated by melatonin treatment in the leaves, 1325 DEGs were expressed in the roots, and only 21 genes were expressed both in the leaves and roots.

Volcano plots were constructed to see how many transcripts were significantly regulated under the different treatments (Figure S2). The significant DEGs are represented by red (upregulated)/green (downregulated) dots with |log_2_ (fold change)| ≥ 1 and a FDR (*q* value) less than 0.05. The results showed that fewer transcripts were regulated by melatonin treatment than by salt treatment.

**Figure 4:**
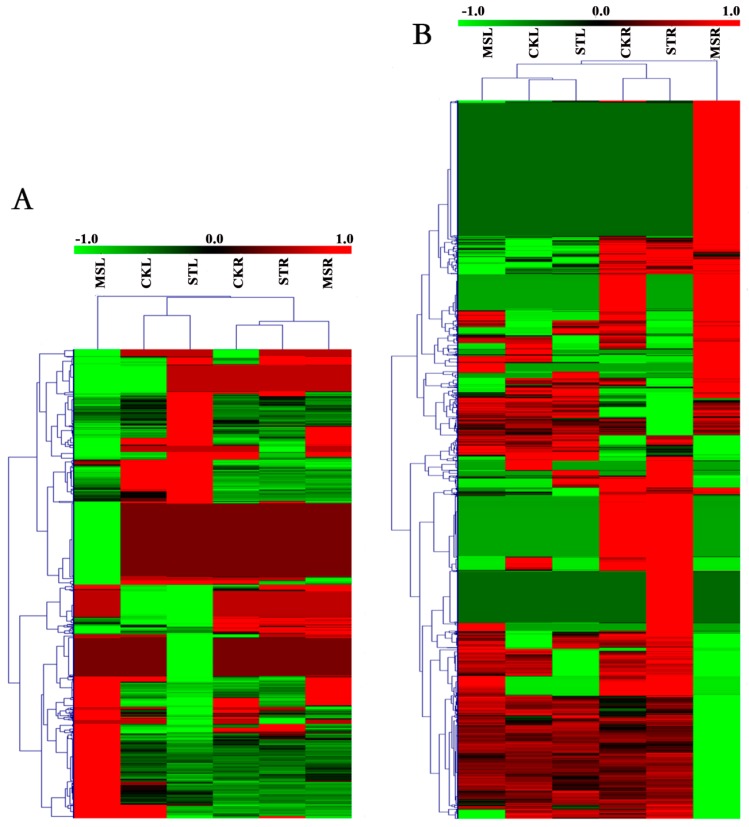
Heat map showing the expression mode and strength of the differentially expressed genes (DEGs) in the different samples. Expression values from RNA-seq data were log_2_-transformed and are displayed as filled blocks colored green to red. (**A**) Expression profiles under different treatments of the DEGs from the MSL versus STL comparison. (**B**) Expression profiles under different treatments of the DEGs from the MSR versus STR comparison.

A total of 880/1799 DEGs in the leaves/roots were identified by RNA-seq between the ST treatment and MS treatment (Table S1). The expression profiles of these DEGs under the different treatments are shown in Figure 4. The upregulated and downregulated genes between the treatments/tissues are indicated by hierarchical clustering analysis.

### 2.5. Gene Ontology (GO) Analysis of Melatonin-Specific DEGs in the Leaves and Roots

With the threshold of *p* < 0.05, the results of the GO analysis of the DEGs between the melatonin and salt stress treatments in the leaves and roots are partly shown in Table 2 (the detailed GO items are listed in Table S2). Three categories, biological processes (BPs), cellular components (CCs), and molecular functions (MFs), composed the GO results. There were 41 and 47 items separately expressed in the BP category in leaves and roots, respectively, in which the first five items in the leaves were ethylene-mediated signaling pathway (GO:0009873), cell redox homeostasis (GO:0045454), response to wounding (GO:0009611), flower development (GO:0009908), and response to stress (GO:0006950), while the first five items in the roots were regulation of transcription, DNA-dependent (GO:0006355), transcription, DNA-dependent (GO:0006351), ethylene-mediated signaling pathway (GO:0009873), positive regulation of transcription, DNA-dependent (GO:0045893), and auxin-mediated signaling pathway (GO:0009734). Three items, DNA-dependent (GO:0006355), response to wounding (GO:0009611), and ethylene-mediated signaling pathway (GO:0009873), existed in both the leaves and roots. Similarly, there were 28 and 42 items separately expressed in the MF category in the leaves and roots, in which the first five items in the leaves were sequence-specific DNA binding transcription factor (TF) activity (GO:0003700), electron carrier activity (GO:0009055), protein disulfide oxidoreductase activity (GO:0015035), pyridoxal phosphate binding (GO:0030170), and receptor serine/threonine kinase binding (GO:0033612), while the first five items in the roots were DNA binding (GO:0003677), sequence-specific DNA binding TF activity (GO:0003700), sequence-specific DNA binding (GO:0043565), protein dimerization activity (GO:0046983), and peroxidase activity (GO:0004601). Two items, DNA binding (GO:0003677) and sequence-specific DNA binding TF activity (GO:0003700), existed in both the leaves and roots. Moreover, the most significant item in the CP category in the leaves and roots was the nucleus (GO:0005634).

### 2.6. Kyoto Encyclopedia of Genes and Genomes (KEGG) Analysis of Melatonin-Driven DEGs in the Leaves and Roots

Four significant KEGG pathways each specifically in the leaves and roots were identified by pathway enrichment analysis (*p* < 0.05) (Table 3). Two pathways, plant hormone signal transduction (ko04075) and diterpenoid biosynthesis (ko00904), were simultaneously significant in both tissues. Among the significant pathways, the pathway of ko04075 had the most DEGs in both leaves and roots (9 and 19 genes, respectively).

### 2.7. Validation of Sequencing Data by Quantitative Real-Time PCR (qPCR) Analysis

**Figure 5:**
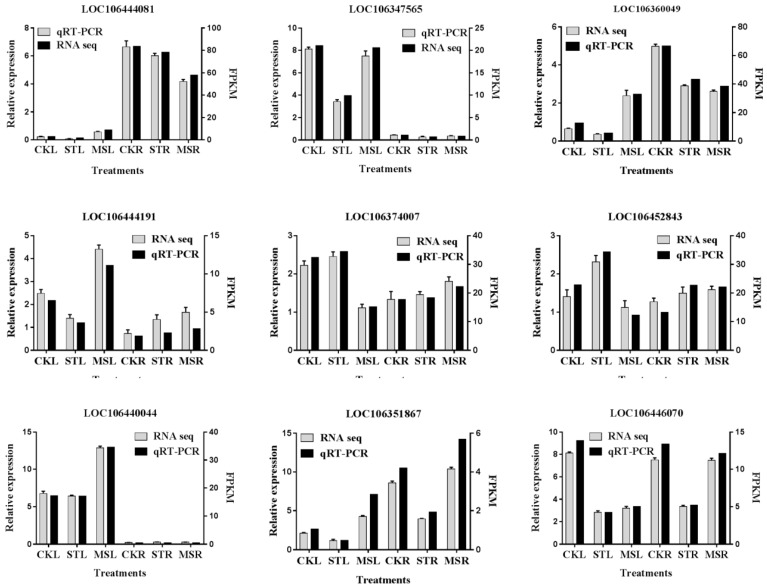
Validation of nine randomly selected genes by Quantitative Real-Time PCR (qRT-PCR). The mRNA expression levels were normalized to the expression level of *ACTIN*, and the means from three biological replicates are shown. The accumulated transcripts of the candidate genes in FPKM (Fragments Per Kilobase of transcript per Million mapped reads, right y-axis) were got from the RNA-seq data.

To validate the expression data from RNA-seq, nine genes with different expression levels according to their FPKM values were selected for real-time RT-PCR analysis. The expression levels of these ten genes under each treatment were confirmed based on the qPCR method. Comparisons of the relative expression levels of these genes evaluated by the FPKM and qPCR methods are shown in Figure 5. Although the change folds were not very similar to the data revealed by transcriptome sequencing, all the candidate genes appeared to have the same expression level tendency as those derived from the DEG data.

### 2.8. Expression of the TFs in Response to MS Treatment

**Figure 6:**
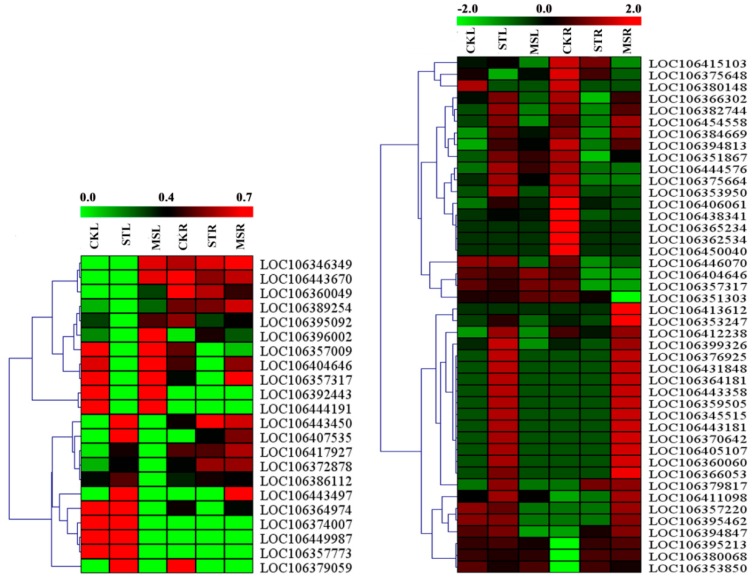
Expression modes of melatonin-specific transcription factor (TF) genes in the leaves and roots. **Left**: In the leaves. **Right**: In the roots. A redder color indicates more transcript accumulation, and greener indicates less. The codes of regulated genes at the right of filled blanks and the detailed explanations of these genes could be found in Table S3.

Genes that code for TFs were filtered from the comparisons between MS and ST in both the leaves and roots (shown in Table S3). Twenty-two TF genes belonging to 9 TF groups were significantly expressed in the leaves, of which 11 genes were upregulated and 11 genes were downregulated (Figure 6). The ERF and MADS-box groups were the most common. Forty-four TF genes belonging to 16 TF groups were significantly expressed in the roots, of which 19 genes were upregulated and 25 genes were downregulated. ERF, WRKY, leucine zipper, and GATA were the most common groups.

## 3. Discussion

### 3.1. Melatonin Improved the Impaired Phenotype of Rapeseed Seedlings Under Salt Stress

It is widely known that salt stress can impair rapeseed seedling growth with harmful symptoms, such as reduced plant height and weight (shoots and roots) and small, dark green leaves [33,34,35,36]. The same salt-induced phenotypes were observed in this study. As a new plant hormone, melatonin has been proven to promote seed germination, enhance plant growth, delay leaf senescence, and improve salt stress tolerance in several agricultural crop species [1,26,27,28]. Here, we found that rapeseed seedlings under salt stress plus melatonin treatment, to a certain extent, had similar macroscopic phenotypes as those under the control treatment. Furthermore, we also reported for the first time that a low-dose application of exogenous melatonin could alleviate injury to rapeseed seedlings induced by salt stress via physiological parameters (such as antioxidant enzymes, H_2_O_2_ and proline content) [32]. Therefore, the recovery of seedling growth proved the function of melatonin, which forecasts the possibility of using melatonin as an activator for promoting rapeseed seedling growth in saline fields in the future.

### 3.2. TF Functions under Melatonin Treatment

TFs have been confirmed to perform vital functions in regulating abiotic stress responses in plants [37]. Here, several families of TFs (mainly ERF, MADS-Box, and WRKY families in the shoots and ERF, WRKY, leucine zipper, GATA, and MADS-Box families in the roots) were identified by comparing the gene expression between the salt treatment and salt plus melatonin treatments, in which the ERF, WRKY, and MADS-Box families were common between whole plants. ERFs specifically bind the GCC-box, which is involved in genes’ ethylene-responsive transcription when plants are faced with environmental stimuli [21,38]. ERFs have been extensively reported to affect the tolerance to salt stress in crops [38,39]. Meanwhile, melatonin has also been confirmed to enhance plant resistance to abiotic stresses (including salt stress), in which modulating the activity of TFs (including ERFs) is one of the pivotal mechanisms [40,41]. Furthermore, the interaction between melatonin and ERFs functioning in resisting stresses has been gradually discovered recently. The expression of ERFs was changed with exogenous melatonin in *Arabidopsis* under stress, implying the associating roles of ERFs with melatonin [42]. *SIERF2* was induced by melatonin and was contributed to postharvest ripening and quality improvement of tomato fruit, showing ERF is a point of melatonin’s functional pathway [43]. Two ERFs (*Cla021070* and *Cla022648*) were proved to combine with melatonin and function under cold treatment [44]. Here, ERF was the most accumulated family of TFs, in which 9 ERF genes in leaves and 15 ERF genes were screened as DEGs, indicating the importance of ERF during melatonin’ function process in rapeseed seedlings facing salt stress. The WRKY family proteins are characterized by a DNA-binding domain consisting of four-sheet strands, forming the WRKY motif with a typical WRKYGQK sequence, followed by a zinc finger motif [37]. WRKYs were shown to regulate melatonin biosynthesis in cassava and the response of melatonin to cold tolerance in *Citrullus lanatus* L [45,46]. MADS domain TFs are involved in controlling many developmental processes in flowering plants, ranging from pollen and embryo sac development to root, flower, and fruit development [47]. We found that the TF genes showed various expression modes regulated by melatonin in response to salt stress. Even the same gene exhibited opposite expression in the leaves and roots. This study confirmed that TFs function with melatonin in the resistance against salt stress. However, few TFs have been demonstrated to cooperate with melatonin to function in response to salt stress in detail [48]. This would be the next step to explore.

### 3.3. Pathways Induced by Melatonin in Response to Salt Stress Treatment

Because too many GO analysis results were enriched in the comparison between the ST and MS treatments, we used the KEGG results to focus on the major melatonin-related pathways and pivotal genes, which would emphasize the main melatonin function.

#### 3.3.1. Two Pathways (ko00100—Steroid Biosynthesis in the Leaves, and ko04075—Plant Hormone Signal Transduction in the Leaves and Roots) Enriched by Melatonin Promoted CS Synthesis, Which May Be One Reason for the Enhanced Seedling Growth

Brassinolides (BRs) are polyhydroxylated steroid hormones that regulate various aspects of plant growth and development by controlling cell division and elongation [49,50,51]. Compared to those in the ST treatment, two BR-related pathways were significantly regulated under the MS treatment: Steroid biosynthesis (Ko00100) in the leaves and plant hormone signal transduction (Ko04075) in both the leaves and roots. The former leads to the synthesis of campesterol (CS), the precursor of brassinolide (BL, the most active BR) [50,52]; the latter includes the BR signal transduction process [53]. Three genes during campesterol synthesis were regulated in response to melatonin. The squalene epoxidase (SQE, EC1.14.14.17, also called squalene monooxygenase) oxidizes the substrate squalene to 2, 3-oxidosqualene, which is then cyclized to cycloartenol in plants. SQE1 has been identified as a fundamental enzyme involved in this biosynthetic step in Arabidopsis [54,55,56]. The *SQE* gene (LOC106348279) was upregulated, which would positively increase the product. Expression of the *sterol methyl-transferase* (*SMT*) gene (LOC106440921), encoding an S-adenosylmethionine-dependent C-24 SMT that catalyzes a single methyl addition during cycloartenol formation, was downregulated, which may affect the products of the following steps. However, this enzyme of the *Arabidopsis* mutant *smt1-1* changed some components of major sterols, implying its effect on BL synthesis [57]. The third enzyme, ∆^7^-sterol-C_5_-desaturase (STE1), catalyzes C_5_-C_6_ desaturation to yield ∆^5^-sterol (LOC106438944). *Arabidopsis ste1* mutants had drastically reduced contents of campesterol, implying the key function of STE1 [57]. The upregulation of the *STE1* gene certainly increases the production of campesterol. We indeed detected decreased CS production under the ST treatment but increased CS production under the MS treatment. Moreover, two genes related to BR signal transduction were both downregulated separately in the leaves and roots. Cyclins are a family of proteins that control the progression of cells through the cell cycle by activating cyclin-dependent kinase (Cdk) enzymes [58]. Plant D-type cyclins (CycD) have been suggested to control both the commitment to cell division and the responses of plant cells to extracellular signals during G1 [59]. Members of the CycD3 group play a role in S-phase entry in response to plant hormones and spatial signals [60,61]. Downregulation of CYCD3;1 is an important factor in the onset of cellular expansion and differentiation in plants. As expected, the *CYCD3* (LOC106444303) gene was downregulated in the leaves in response to melatonin. Brassinosteroids bind to the extracellular domain of the receptor kinase BRASSINOSTEROID INSENSITIVE 1 (BRI1) to activate a signal transduction cascade that regulates nuclear gene expression and plant development [62,63]. Two substrates could be activated by BRI1: One is BRI1 KINASE INHIBITOR 1 (BKI1), which acts as a positive regulator by binding to a subset of 14-3-3 proteins; the other is BSK (BR-signaling kinase), which then interacts with BSU1 to dephosphorylate and inactivate BIN2. Upon inactivation of BIN2, BZR1, and BZR2/BES1 could undergo dephosphorylation, accumulate in the nucleus and regulate the expression of BR-responsive genes. We found that the *BSK* gene (LOC106446564) was down-regulated in the roots under melatonin treatment. There are two reasons for this: One involves promoting the function of the BKI1 branch, and the other is probably that there is another mechanism mediated by BSKs in the complex process by which BRs function.

#### 3.3.2. The JA Synthesis Pathway (ko00592—Alpha-Linolenic Acid Metabolism) in Leaves Was Inhibited by Melatonin

JA and its methyl ester (MeJA), collectively named jasmonates (JAs), are lipid-derived signals and have been demonstrated to be involved in plant growth-inhibiting and senescence-promoting activity [64]. JA was firstly suggested to be involved in the plant salinity response [65]. The JA synthesis process (α-linolenic acid metabolism, Ko00592) and the JA signal transduction process (plant hormone signal transduction, Ko04075) were significantly enriched during melatonin treatment. The expression of two genes, the *hydroperoxide lyase* (*HPL1*) gene (LOC10644044) and the *allene oxide cyclase* (AOC) gene (LOC106443552 and LOC106352449), was significantly regulated in the former pathway. AOC and HPL are located on the branches of the oxylipin pathway; these enzymes underlie the production of JAs and aldehydes (green leaf volatiles, GLVs), respectively [66]. Upregulation of *HPL1* and downregulation of *AOC* steered the flow of synthesis to GLVs, which potentially decreased JA production. Salt stress was demonstrated to induce JA synthesis; however, melatonin reversed this tendency and lost JA inhibition during plant development [65]. Additionally, JASMONATE ZIM-DOMAIN (JAZ) proteins, which possess a 28-amino acid conserved domain (ZIM domain), are important for delivering the JA signal. JAZs bind to bHLH TFs (e.g., MYC2, MYC3, MYC4 and MYC5) that are activators of JA responses by repressing their transcriptional activity and deactivating the expression of early JA-responsive genes [67]. Increased *JAZ* (LOC106354531) expression in the roots under melatonin treatment would bind more MYCs, leading to less activity on the downstream JA-responsive genes. In this experiment, salt stress up-regulated the production of JA, inhibiting the growth of rapeseed seedlings; however, adding melatonin down-regulated the production of JA, benefiting seedling recovery.

#### 3.3.3. The GA Synthesis Pathway (Ko00904—Diterpenoid Biosynthesis) Was Activated in the Roots

GAs form a large family of diterpenoid compounds, some of which are bioactive growth regulators that control various developmental processes, including stem elongation, leaf expansion, trichome development, and so forth [68]. The major bioactive GAs, which include GA1, GA3, GA4, and GA7, are derived from a basic diterpenoid carboxylic acid skeleton and commonly have a C3-hydroxyl group [69]. The biosynthesis of GA (Ko00904) in higher plants can be divided into three stages: (1) biosynthesis of *ent*-kaurene in proplastids; (2) conversion of *ent*-kaurene to GA_12_ via microsomal cytochrome P450 monooxygenases; and (3) formation of C_20_- and C_19_-GAs in the cytoplasm. The expression of three genes involved in the GA synthesis process was significantly regulated. The first one (LOC106442380), in the leaves, encodes gibberellin 20-oxidase (GA20ox), which catalyzes the oxidation and elimination of carbon-20, yielding C19-GAs. Regulation of 20-oxidase gene expression was shown to affect the level of endogenous GAs and influence plant growth [70]. The second gene (LOC106398539), in the leaves, encodes the key enzyme gibberellin 3-beta-dioxygenase (GA3ox), which catalyzes the conversion of inactive to bioactive GAs. The third gene (LOC106357323), in the roots, encodes gibberellin 2-oxidase (GA2ox). Regulation of the expression patterns of these three genes would increase GA3 production. An increase in GA was detected in the leaves under the salt plus melatonin treatment, although the level of GA was not up to the level of control. The *GIBBERELLIN INSENSITIVE DWARF 1* (*GID1*) genes (LOC106413374 and LOC106453059) in the gibberellin signal transduction pathway were strongly upregulated in the leaves and roots in response to melatonin treatment. GID1 was identified as the soluble GA receptor that interacts with GA and DELLA proteins to form a complex that inhibits the repression activity of DELLAs on GA signaling [71]. The relatively high yield of GA and relatively strong GA signal transduction undoubtedly promoted seedling growth.

#### 3.3.4. Genes Regulated by Melatonin in the Ko04075 Pathway Potentially Led to Enhanced Signal Transduction of Several Plant Hormones (Auxin, Cytokinin and Abscisic Acid) in Both the Leaves and Roots

Auxin acts as a general coordinator of plant growth and regulates transcription via an elegantly short signal transduction pathway [72]. In brief, auxin brings together members of the TRANSPORT INHIBITOR RESPONSE1/AUXIN SIGNALING F-BOX (TIR1/AFB) family and the Aux/IAA transcriptional repressor (AUX/IAA) family, leading to TIR1/AFB degradation by ubiquitination. The AUXIN RESPONSE FACTOR (ARF), which binds Aux/IAA, is then released and can act as a transcriptional activator of auxin-regulated genes. Small auxin upregulated RNAs (SAURs) are key effector output genes that can promote cell expansion/division and activate plant growth and development [73]. Melatonin regulated the expression of *TIR1* (in the roots), *Aux/IAA* (in the leaves and roots) and *SAUR* (in the leaves and roots) genes, which may accelerate vegetative growth by cell enlargement.

Cytokinins are implicated in nearly all aspects of plant growth and development, including cell division, shoots initiation and development, the light response, and leaf senescence [74]. In higher plants, the cytokinin signaling mechanism is a two-component system (TCS) that involves three different types of proteins: Histidine kinases (AHKs), histidine phosphotransfer proteins (AHPs), and response regulators (ARRs) [75]. By acting as negative regulators, type-A response regulators (A-ARR) may mediate downstream activity in the two-component cytokinin signaling pathway, while type B response regulators (B-ARR) are TFs that act as positive regulators. In *Arabidopsis*, A-ARR and B-ARR were demonstrated to affect root elongation [76]. The expression of three genes in the roots homologous to *ARR4* (*A-ARR*), *ARR7* (A-ARR), and *ARR12* (B-ARR) was down-regulated under melatonin treatment, which may benefit rapeseed seedling growth.

Abscisic acid (ABA) is an endogenous small-molecule growth inhibitor and regulator of plant stress physiology [77]. ABA binds to the receptor PYRABACTIN RESISTANCE1 (PYR1)/PYR1-LIKE (PYR/PYL) to form a complex and inhibits the activity of phosphatases type-2C (PP2C) proteins, which negatively regulate ABA signaling via repression of sucrose nonfermenting 1-related protein kinase 2s (SnRK2s), which are positive regulators of downstream targets [78]. Up regulation of *PYR/PYL* expression and down regulation of PP2C expression in the roots led to weakened ABA signal, which may alleviate the root growth inhibition by ABA.

#### 3.3.5. Two Pathways (ko00940 and Ko00780) Related to Lignin Synthesis and Fatty Acid Synthesis Responded to Melatonin in the Roots

Lignin is one of the most important secondary metabolites produced by the phenylalanine/tyrosine metabolic pathway (Ko00940) in plant cells [79]. Lignin and its related metabolism play important roles in the growth and development of plants and in response to various environmental stresses [80]. In lignin biosynthesis, caffeic acid-O-methyltransferase (COMT) is primarily responsible for catalyzing the *O*-methylation of the 5-hydroxyl group of 5-hydroxyconiferaldehyde to produce sinapaldehyde. Peroxidases (PODs) and lactases are two key enzymes that participate in the polymerization of monomers. Glycosylation of monolignols is catalyzed by UDP-glycosyltransferases (UGTs) belonging to the glycosyltransferase (GT) family. Up-regulation by melatonin of the expression of the *COMT*, *POD*, and *UGT* genes steers the flow in favor of lignin synthesis, which increases the resistance to salt stress.

Fatty acid synthesis has been demonstrated to be intimately related to salt tolerance because the fatty acids are the key components of plant cell membranes, which house Na^+^ transport channels [81]. Fatty acids (especially unsaturated acids) play an essential role in the biophysical characteristics of cell membranes and determine the proper function of membrane-attached proteins. Membrane integrity and function, which are determined by structure and fluidity, are largely affected by lipid composition and the degree of fatty acid desaturation in plants and other organisms. The expression of three vital genes (*FabG*, *FabZ*, and *Fabi*) in the pathway (Ko00780), which encode the 3-oxoacyl-[acyl-carrier protein] reductase [EC: 1.1.1.100] 3-hydroxyacyl-[acyl-carrier-protein] dehydratase [EC: 4.2.1.59] and enoyl-[acyl-carrier protein] reductase I [EC: 1.3.1.9 1.3.1.10], was down-regulated by melatonin treatment [82].

### 3.4. Proposed Model of Melatonin in Alleviating the Harm from Salt Stress

**Figure 7:**
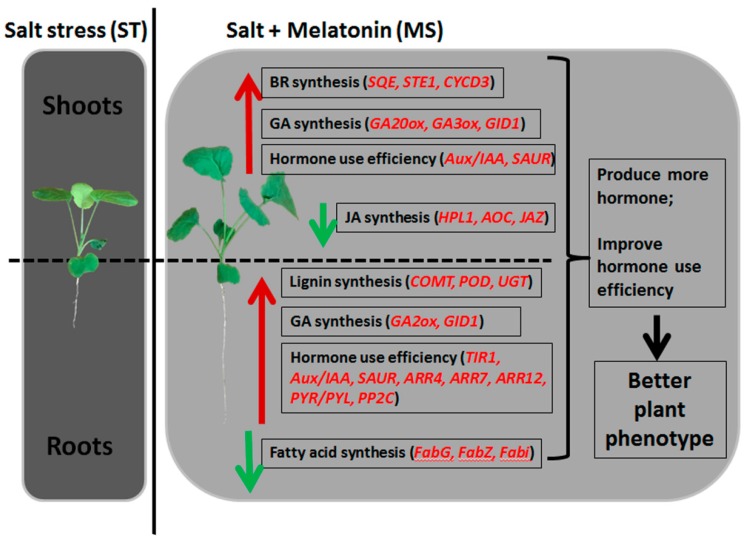
Proposed model for the function of melatonin in alleviating the harm from salt stress. The up-pointing red arrows mean that the candidate pathways are enhanced; the down-pointing green arrows mean that the candidate pathways are diminished. The main affected pathways were shown in the boxes. The vital genes affecting the corresponding pathways are shown in red italics within brackets. BR = brassinolides; GA = gibberellic acid; JA = jasmonic acid.

In this study, salt stress-treated rapeseed seedlings were smaller, implying inhibition from salt treatment. However, the growth of seedlings under the salt plus melatonin treatment recovered, and their biomass was similar to that under the control, proving that melatonin could alleviate the harm from salt stress and promote seedling growth. Moreover, increased amounts of several plant hormones were detected in the leaves of seedlings, showing the potential relationship between traditional plant hormones (CS, JA, and GA) and melatonin. Our results suggested the existence of several putative mechanisms to explain the recovery growth when melatonin was applied during salt stress. (1) Melatonin activated the synthesis of hormones. With the addition of melatonin, expression of the key genes responsible for the synthesis of CS, JA, and GA was upregulated. The synthesis of CS and GA increased to promote seedling growth, while that of JA was reduced to decease the inhibition of seedling growth. (2) Melatonin helps to increase hormone use efficiency. Genes in the branched pathways of plant hormone signal transduction were expressed, and high levels of IAA, CTK, and ABA were beneficial, leading to seedling growth. (3) In the roots, melatonin triggered two other pathways because the roots were directly exposed to salt stress. Overall, though salt stress inhibited rapeseed seedling growth, melatonin could alleviate this effect and could help restore seedling development by regulating plant hormone production and promoting their function (Figure 7). The results of our study indicated the possible application of melatonin in crop production on saline soils.

## 4. Materials and Methods

### 4.1. Plant Material and Treatments

The rapeseed variety Zhongshuang No. 11 (ZS11), which was supplied by the Oil Crops Research Institute (CORI) of the Chinese Academy of Agriculture Sciences, was used in this study. Seedlings were cultivated according to the method described [17]. Uniform seedlings at the two-leaf stage were randomly chosen and transplanted into new plastic boxes filled with three different solutions: (1) fresh Hoagland’s solution (set as the control, CK); (2) fresh Hoagland’s solution with 125 mM NaCl (set as salt stress, ST); and (3) fresh Hoagland’s solution with 125 mM NaCl plus 50 µM melatonin (set as the melatonin plus salt treatment, MS). The concentrations of NaCl and melatonin were determined according to previous results [17]. The whole procedures were replicated three times. After 7 d of treatment, the leaves (L) and roots (R) were separately sampled from 5 individuals under each treatment from each replicate (18 samples in total) and quickly stored individually in liquid N_2_. All the treatments were performed in a growth chamber (24 °C, 16/8 h light/dark and 60–80% humidity).

### 4.2. Determination of Physiological Parameters and Hormone Contents

After measuring the plant height, the uniform seedlings of each replicate under the CK, ST and MS treatments were split into two parts, shoots and roots, whose fresh weights were measured. Three plant hormones (campesterol (CS), jasmonic acid (JA), and gibberellic acid 3 (GA3)) were measured via HPLC-MS/MS analysis as previously described [83,84].

### 4.3. RNA Extraction, cDNA Library Construction, Illumina Sequencing, and Sequence Analysis

The RNA of all 18 samples was extracted according to the instructions of a total RNA purification kit (TRK1001, LC Science, TX, USA). RNA quality was determined by a Bioanalyzer 2100 and RNA 6000 Nano Lab Chip Kit (Agilent, Santa Clara, CA, USA). Approximately 50 ng of each purified RNA was used to construct libraries following the detailed steps of a gene expression sample prep kit (Illumina, San Diego, CA, USA), which included mRNA purification, mRNA fragmentation, adding adapters, reverse transcription and library validation. The 18 gene expression libraries were named CKL-1, CKL-2, CKL-3, CKR-1, CKR-2, CKR-3, STL-1, STL-2, STL-3, STR-1, STR-2, STR-3, MSL-1, MSL-2, MSL-3, MSR-1, MSR-2, and MSR-3 (L refers to leaves, and R refers to roots). After deep sequencing on an Illumina HiSeq 4000 platform at LC-Bio Company (Hangzhou, China), these digital sequence data were transformed into raw nucleotide reads with a 150 bp length. Via quality-control steps, the remaining sequences were considered valid reads (all of the data were uploaded to the Sequence Read Archive (SRA) database of the National Center for Biotechnology Information (NCBI) along with their ID PRJNA561674) to perform subsequent analyses.

### 4.4. Read Mapping, Confirmation of Differentially Expressed Genes, and Gene Expression Profiles

The valid data from each sample library were mapped to the reference database of the *Brassica napus* genome (http://plants.ensembl.org/Brassica napus/Info/Index) using Bowtie2 software (Johns Hopkins University, Baltimore, Maryland, USA) to count the mapping events on both the sense and antisense strands. The expression level of each gene was calculated using the FPKM method [85]. The specific gene expression strength was recorded as the average of three replicates. The differentially expressed genes (DEGs) were identified by Cufflinks software by setting the thresholds of *q* value ≤ 0.05 and a |log_2_(FPKM) ratio| ≥ 1 as significant gene expression differences between samples. The *q* value is a revised *p* value for estimating the false discovery rate (FDR) among multiple samples [86]. Specifically, gene expression comparisons of the samples with different treatments (STL/CKL, MSL/STL, STR/CKR, MSR/STR) were performed. Heat maps showing the expression patterns of the DEGs between the treatments were produced by Mev software (J. Craig Venter Institute, La Jolla, CA, USA).

### 4.5. Gene Ontology (GO) Functional Enrichment Analysis

All DEGs were used to query the GO database (http://www.geneontology.org/) via BLAST by employing Blast2GO software (Biobam, Valencia, Spain). The gene numbers for each term of three ontologies (molecular function, cellular component and biological process) were calculated using a hypergeometric test, and the enrichment significance of each GO term was decided by the *p* value, which was calculated according to the described methods [87]. We used *p* ≤ 0.05 as the threshold for judging significantly enriched GO terms.

### 4.6. Kyoto Encyclopedia of Genes and Genomes (KEGG) Enrichment Analysis

Blast2GO software and the significance formula (*p* value) for GO analysis were again used to query the DEGs against the KEGG database (http://www.genome.jp/) via BLAST. The most enriched KEGG was enlisted in order according to the *p* value. A *p* value < 0.05 was required for differences to be considered statistically significant.

### 4.7. Quantitative Real-Time PCR (qPCR) Analysis

The RNAs from each replicate of each treatment were mixed equally as a sample of the certain treatment. The RNA samples were then used as templates for performing cDNA synthesis after the genomic DNA erasing step. The primers used and the corresponding genes are listed in Table S4. The expression level of the *β*-actin gene was used as a control. The qPCR process was conducted on an ABI 7500 Real-time PCR platform. The ΔΔC_t_ value of each gene was calculated via Microsoft Excel to compare the expression levels between two treatments.

## Figures and Tables

**Table 1:** Statistic of sequence data.

	CKL	CKR	STL	STR	MSL	MSR
Total reads	63,336,621	63,759,278	45,003,114	51,101,209	48,888,169	54,514,887
Base (G)	9.5	9.56	6.75	7.66	7.33	8.18
Valid reads	62,330,568	62,915,811	48,308,551	53,679,387	44,428,598	50,153,037
Mapped reads	44,355,294	40,347,532	34,248,178	37,128,797	31,439,376	30,284,470
	(71.15) *	(64.09)	(70.91)	(69.15)	(70.72)	(60.54)
Unique Mapped reads	36,308,254	34,150,965	27,843,516	31,419,760	25,251,723	25,677,221
	(58.24)	(54.25)	(57.66)	(58.52)	(56.71)	(51.33)
Multi Mapped reads	8,047,041	6,196,566	6,404,662	5,709,037	6,187,653	4,607,249
	(12.91)	(9.84)	(13.25)	(10.63)	(14.01)	(9.21)
Genes	44,404	47,647	42,780	47,200	39,527	42,338

* meant the percent of the corresponding data of the total valid data. STL = leaves in ST treatment; CKL = leaves in CK treatment; MSL = leaves in MS treatment; STR = roots in ST treatment; CKR = roots in CK treatment; MSR = roots in MS treatment.

**Table 2:** Gene Ontology (GO) classifications of DEGs in the leaves and roots. The genes were divided into three main categories by GO analysis: Biological process, molecular function, and cellular component. Numbers indicate the regulated genes in the corresponding sub-categories.

Category	MSL vs. STL		MSR vs. STR	
	Items	Number of Genes	Items	Number of Genes
Biological process	ethylene mediated signaling pathway	10	regulation of transcription, DNA-dependent	46
	cell redox homeostasis	7	transcription, DNA-dependent	43
	response to wounding	6	ethylene mediated signaling pathway	18
	flower development	6	positive regulation of transcription, DNA-dependent	11
	response to stress	6	auxin mediated signaling pathway	11
	secondary cell wall biogenesis	4	response to wounding	10
	glucosinolate catabolic process	3	negative regulation of defense response	6
	cell-cell signaling involved in cell fate commitment	3	cytokinin mediated signaling pathway	6
	gibberellic acid mediated signaling pathway	3	hydrogen peroxide catabolic process	6
	response to biotic stimulus	3	negative regulation of abscisic acid mediated signaling pathway	4
	response to red light	3	defense response to fungus, incompatible interaction	4
	plant-type cell wall organization	3	hyperosmotic salinity response	4
	response to gibberellin stimulus	3	cellular response to heat	3
Cellular component	nucleus	65	nucleus	119
	apoplast	11	extracellular region	27
	extracellular space	4	ER body	2
	chloroplast membrane	4	extrinsic to plastid membrane	1
	primary cell wall	2	P granule	1
Molecular function	DNA binding transcription factor activity	22	DNA binding	54
	electron carrier activity	12	sequence-specific DNA binding transcription factor activity	44
	protein disulfide oxidoreductase activity	7	sequence-specific DNA binding	26
	pyridoxal phosphate binding	5	protein dimerization activity	13
	receptor serine/threonine kinase binding	3	peroxidase activity	8
	two-component response regulator activity	3	drug transmembrane transporter activity	6
	2 iron, 2 sulfur cluster binding	3	acid phosphatase activity	4
	phosphorylase activity	2	caffeate O-methyltransferase activity	3
	brassinosteroid sulfotransferase activity	2	protein serine/threonine/tyrosine kinase activity	3
	allene-oxide cyclase activity	2	AT DNA binding	3
	serine-type endopeptidase inhibitor activity	2	CTP:2-trans,-6-trans-farnesol kinase activity	2
	lyase activity	2	geranylgeraniol kinase activity	2
	water channel activity	2	geraniol kinase activity	2

**Table 3:** List of significant pathways identified by Kyoto Encyclopedia of Genes and Genomes (KEGG) enrichment analysis of DEGs in the leaves and roots. Note: The common pathways were in bold.

Tissues	KEGG ID	Pathway	Number of Transcripts	*p* Value
Leaves	**ko04075**	Plant hormone signal transduction *	9	0.0051
	ko00100	Steroid biosynthesis	3	0.0060
	ko00592	alpha-Linolenic acid metabolism	3	0.0129
	**ko00904**	**Diterpenoid biosynthesis**	2	0.0160
Roots	**ko04075**	**Plant hormone signal transduction**	19	0.0001
	ko00940	Phenylpropanoid biosynthesis	12	0.0014
	ko00780	Biotin metabolism	3	0.0099
	**ko00904**	**Diterpenoid biosynthesis**	3	0.0106

* the common pathways between leaves and roots.

## Supplementary Materials

Supplementary materials can be found at https://www.mdpi.com/1422-0067/20/21/5355/s1. Table S1. DEGs between the ST and MS treatments in the leaves and roots. Table S2. GO analysis of DEGs between the leaves and roots. Table S3. Transcription factors (TFs) that respond to melatonin treatment in the leaves and roots. Table S4. Q-PCR primers used for the detection of genes. Figure S1. Venn diagram showing the number of unique and common DEGs among the comparisons of the different treatments. Values in the brackets showed the percentages of the corresponding gene numbers to total regulated genes. Figure S2. Volcano plots of the transcriptomes between different treatment samples. In the volcano plots, statistical significance (log_10_(*p* value); Y-axis) is plotted against the log_2_(fold change) (*x*-axis). Red and green dots represent transcripts expressed at significantly higher or lower levels in the different comparisons, respectively.
