# Supplementary figures and images for: Melatonin-Induced Transcriptome Variation of Rapeseed Seedlings under Salt Stress
Source: Int J Mol Sci. 2019 Oct 28;20(21):5355. doi: 10.3390/ijms20215355 (PMC6862158; doi:10.3390/ijms20215355)

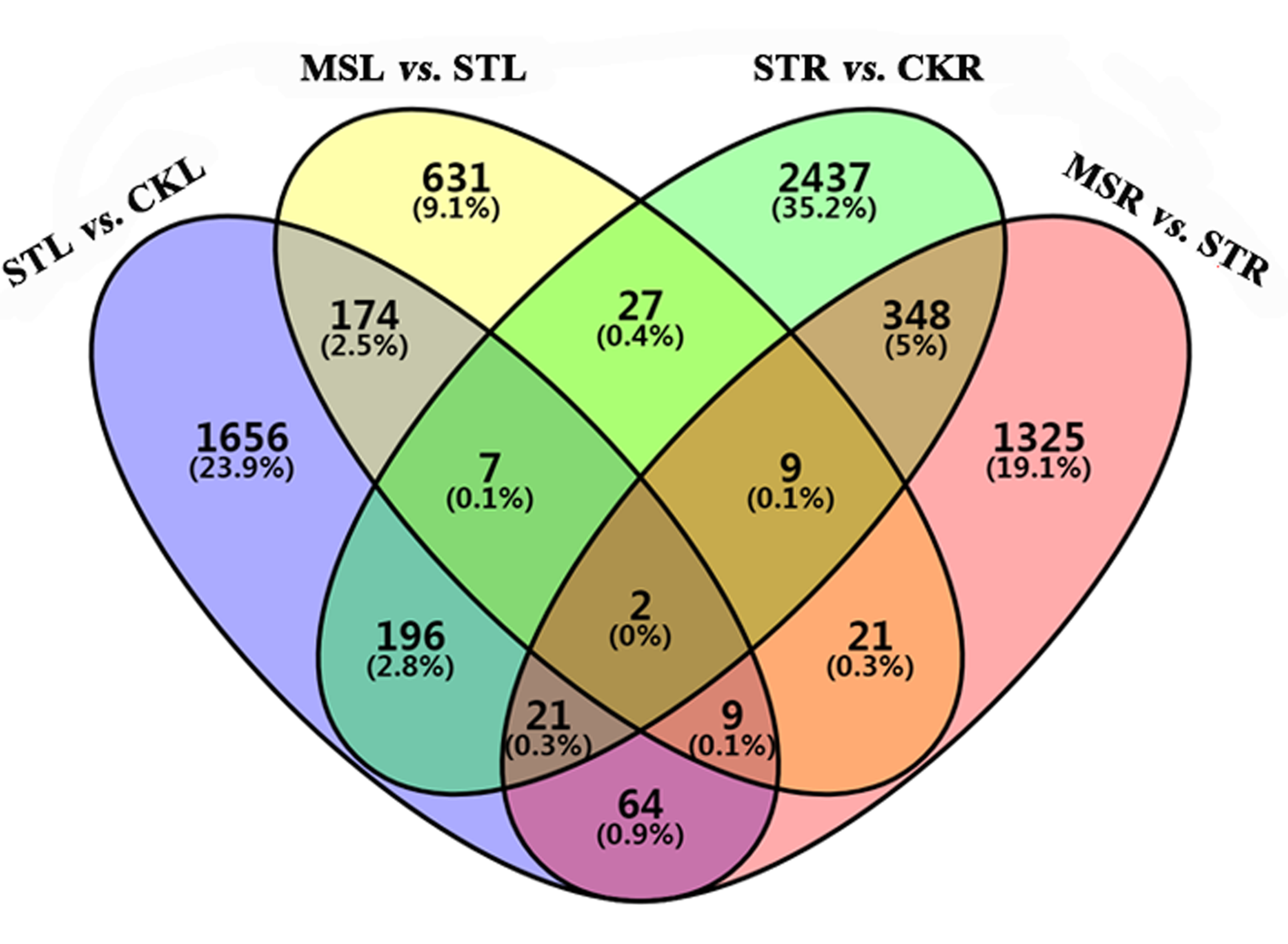

Supplement: Supplementary file 1 [file ijms-20-05355-s001.zip › ijms-601496-supplementary/Figure S1.tif]

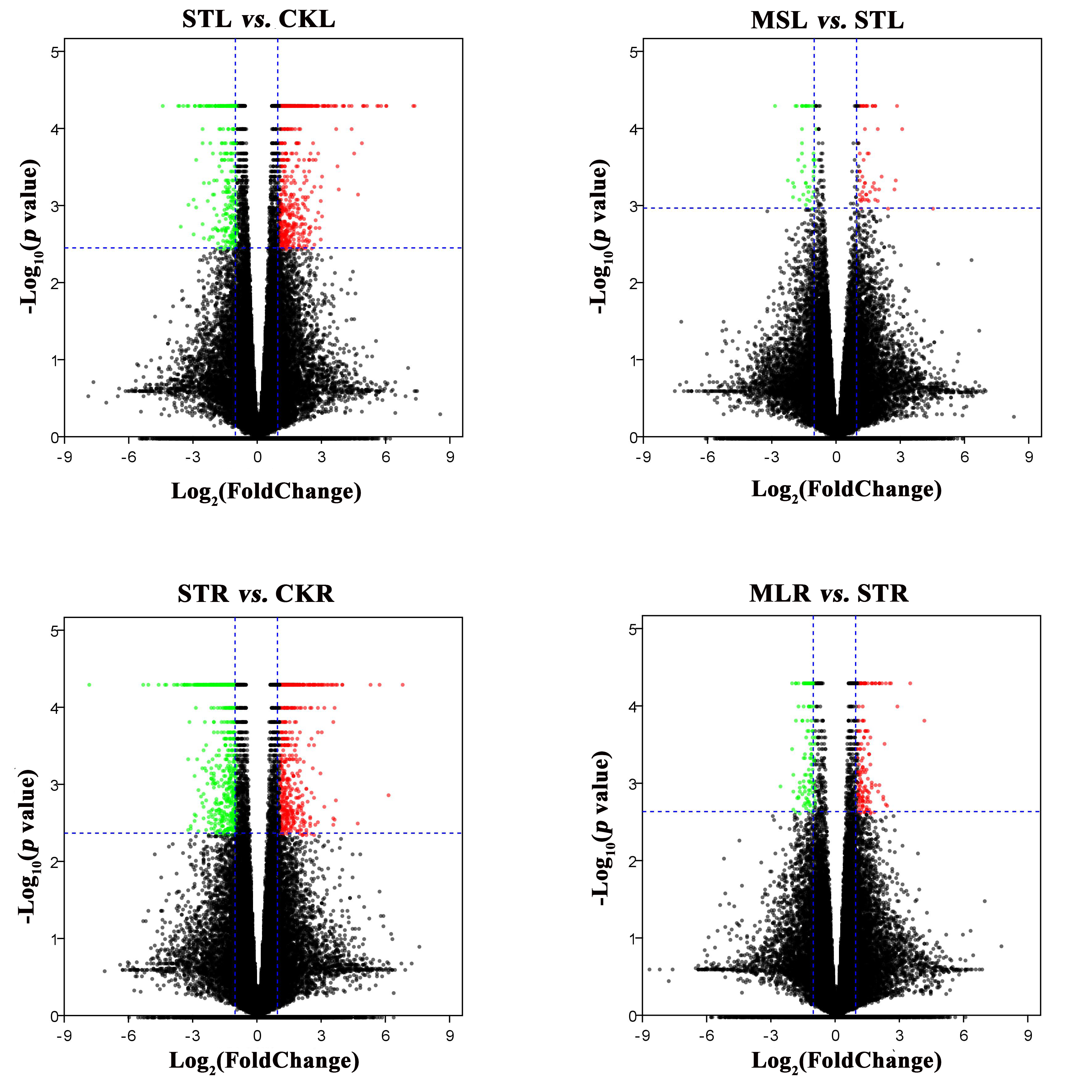

Supplement: Supplementary file 1 [file ijms-20-05355-s001.zip › ijms-601496-supplementary/Figure S2.tif]
